# Effectiveness of Theobromine on Enamel Remineralization: A Comparative In-vitro Study

**DOI:** 10.7759/cureus.5686

**Published:** 2019-09-17

**Authors:** Parvathy Premnath, Joseph John, Nithin Manchery, Gireesh K Subbiah, Nagappan Nagappan, Prabhu Subramani

**Affiliations:** 1 Public Health Dentistry, Asan Memorial Dental College and Hospital, Chengalpattu, IND; 2 Public Health Dentistry, Saveetha Dental College, Saveetha University, Chennai, IND; 3 Oral Health Centre, The University of Queensland, Brisbane, AUS; 4 National Institute for Research In Tuberculosis, Indian Council of Medical Research, Chennai, IND; 5 Public Health Dentistry, Chettinad Dental College & Research Institute, Kanchipuram, IND

**Keywords:** theobromine, theobromine, enamel, enamel, fluoride, fluoride, remineralization, remineralization, tricalcium phosphate, tricalcium phosphate

## Abstract

Background

Remineralizing agents demonstrate potential to reverse early carious lesions. Theobromine containing dentifrices claim to remineralize enamel lesions effectively. The aim of this in-vitro study was to evaluate and compare the remineralization potential of dentifrices containing theobromine, 0.21% sodium fluoride (NaF) with functionalized tricalcium phosphate (f-TCP) and amine fluoride on artificial enamel caries.

Materials and methods

Sound extracted human premolars were demineralized to produce deep artificial carious lesions. The teeth were sectioned longitudinally and allocated to three treatment groups with nine specimens in each group: Group A (NaF + f-TCP), Group B (amine fluoride), and Group C (theobromine). The specimens were then subjected to pH cycling for seven days. Confocal laser scanning microscopy (CLSM) was utilized to record the patterns of demineralization and remineralization. One-way ANOVA and paired t-test were used to analyze changes in lesion depth. The level of significance was set at p<0.05.

Results

All three dentifrices effectively remineralized artificial carious lesions (paired t-test, p<0.001). Of the groups, Group A (54.97%) reported the highest percentage change in lesion depth values followed closely by Group B (51.51%) and Group C (31.71%), respectively.

Conclusion

Within this in-vitro study, theobromine containing dentifrice was effective in remineralizing lesions of enamel. However, theobromine demonstrated less remineralization potential in comparison to dentifrices containing NaF + f-(TCP) and amine fluoride.

## Introduction

Dental caries is often described as a complex, irreversible disease resulting from the dissolution and destruction of calcified tissues of the teeth [1]. Globally, its burden is ever-increasing and frequency pre-eminent as compared to other common diseases of the oral cavity. The disease, furthermore, has a considerable impact on the general health and social and economic well-being of an individual [2]. Prevention of this ubiquitous disease poses a significant and formidable challenge for oral health professionals even in this era of advanced dentistry.

It is well-understood that the caries process involves a constant shift in the equilibrium of tooth minerals, resulting in demineralization (mineral loss) or remineralization (mineral gain) [3]. Therefore, the focus of dental research lately has been on the development of effective methods to facilitate the natural loss of mineral constituents incurred during demineralization. Remineralizing agents provide a supersaturated environment, promoting mineral gain, and are recognized as a viable non-invasive method for the effective management of early carious lesions [4].

A plethora of remineralization agents is available, of which fluoride is the most popular and widely used. The diminished rates of dental caries in several countries today can be attributed to the larger use of fluoride-containing dentifrices [5]. The current theories of mechanism indicate fluorides work primarily via topical mechanisms by inhibiting demineralization and amplifying remineralization by the deposition of fluorapatite crystals in the presence of calcium and phosphate ions [6]. Studies also demonstrate the significant remineralizing efficacy of various concentrations of fluoride-containing dentifrices in arresting caries [7-9].

Nevertheless, concerns have been expressed on the harmful effects of the long-term use of fluoride-containing dentifrices [5]. Furthermore, the ability of fluoride to inhibit caries formation is controlled by the availability of calcium and phosphate ions present in saliva and plaque fluid. Therefore, in conditions like salivary function disorders, the rate of remineralization is affected [10]. Though fluoride remains the cornerstone of non-invasive caries management, dental research has constantly engineered newer non-toxic, anti-cariogenic materials for alternative use.

Theobromine (3,7-dimethylxanthine) is a primary alkaloid derived from the cacao plant. It is a water-soluble, crystalline, bitter powder found in chocolates along with tea and other foods. The consumption of chocolate, a food processed from cacao seeds, in any form, has generally been associated with the increased formation of dental caries [11]. However, in recent years, arguments have been made for the use of theobromine as an effective remineralizing agent and as a possible alternative to fluorides [12-13]. It is believed theobromine, in the presence of calcium and phosphate, forms hydroxyapatite crystallites of an increased size that strengthen the enamel, making it less susceptible to acid attack, which eventually leads to cavitation [12]. Further, results from studies also indicate cocoa bean husk being effective in reducing mutant streptococci [14] and exhibit low toxicity in contrast to fluoride [15]. Toothpaste containing theobromine are commercially available and are used widely in the US, but they are comparatively less popular than other available remineralizing agents worldwide. Moreover, there exists limited evidence to substantiate claims by manufacturers on the remineralization efficacy of theobromine-containing dentifrices and very little is understood of how theobromine compares to other standard remineralizing agents. Thus, the aim of this in-vitro study is to compare and evaluate the remineralization potential of dentifrices containing theobromine, 0.21% sodium fluoride (NaF), with functionalized tricalcium phosphate (f-TCP) and amine fluoride on artificial enamel caries using confocal laser scanning microscopy (CLSM).

## Materials and methods

This in-vitro experimental study was designed and conducted in the Department of Public Health Dentistry, Saveetha Dental College and Hospital. The Scientific Review Board at Saveetha University approved the study protocol.

Sample selection and preparation

Sample size calculations were based on mean differences in lesion depth from the study by Amaechi et al. and were estimated as nine per group (90% power and 5 % α-error).

Sound maxillary pre-molars indicated for orthodontic extractions were collected for this study. All teeth obtained were thoroughly cleaned for soft-tissue debris/calculus and stored in a 10% formalin solution until further use. Each individual tooth was then subjected to the preparation of a treatment window, 1 mm × 1 mm wide, on the buccal surface. The remaining surfaces were coated with acid-resistant nail paint (Lakme, Hindustan Unilever, India). This was done to facilitate lesion depth measurement compared to the unaffected area covered by nail paint.

Preparation of demineralizing and remineralizing solutions

High-grade analytical chemicals and deionized water were used to prepare the respective de- and remineralizing solutions. Demineralizing (DML) solutions were produced by using a mixture of 2.2 mM calcium chloride, 2.2 mM sodium phosphate, and 0.05 M acetic acid; with 1 M potassium hydroxide to adjust the pH to 4.4. The remineralizing (RML) solution consisted of 1.5 mM calcium chloride, 0.9 mM sodium hydrogen phosphate, and 0.15 potassium chloride at a pH adjusted to 7.0 with 1M potassium hydroxide [16].

Dentifrices tested

The following three dentifrices were used for comparison:

· Clinpro™ (3M ESPE, Canada), 0.21% NaF dentifrice with f-TCP

· AMFLOR (Group Pharmaceuticals Ltd., India), 1450-ppm amine fluoride-containing dentifrice

· THEODENT (Theodent classic®; Theocorp Holding Company, Metairie, LA, USA), dentifrice containing a delicate blend of theobromine (cacao extract), calcium, and phosphate

Preparation of Artificial Carious Lesion

All the specimens were then placed into the DML solution (10 ml solution per tooth) for a period of 96 hours. This was done to create artificial carious lesions of approximately 200 to 250 µm deep among the selected teeth. After 96 hours, the teeth were subjected to sectioning. Using a hard tissue microtome (Leica SP 1600, Bensheim, Germany), longitudinal sections were produced, approximately 200 to 250 µm thick. The 27 sections were then divided into three equal groups and allocated systematically. Group A for NaF + f-TCP-containing dentifrice consisted of specimens numbered 1 to 9, Group B for amine fluoride consisted of specimens numbered 10 to 18, and, lastly, Group C (theobromine) included specimens numbered 19 to 27.

Dentifrice Preparation

Toothpaste supernatant was obtained by mixing the respective dentifrice (12 g) with deionized water (36 Ml) in a 1:3 dilution. The suspensions were produced by mechanical agitations (stirring rod) for a minute, followed by centrifuging at 3600 rpm for 20 minutes at room temperature. 

pH Cycling Model

The specimens were placed in an orbital shaker (ThermoFisher Scientific, MA, US) for seven days. The solutions (DML, RML, and toothpaste supernatant) required for each cycle were freshly prepared and distinct containers used for each group during the entire course of the experiment. Prior to the start of the cycle, the pH of the solutions was measured and standardized. A single cycle consisted of alternating episodes of DML twice daily (3 h) with RML (2 h) in between. The groups (A, B, and C) were also subjected to 60s with toothpaste supernatant (5 ml/section) before the first and both before and after the second DML cycles [17].

Evaluation Technique 

CLSM (CLS Leica TCS SL inverted microscope M, Leica Microsystems, Wetzlar, Germany) was used to make pre-treatment and post-treatment records. A 0.1 mM Rhodamine B solution was used to stain the sections for 1 hr to ensure that it does not penetrate through the sound tooth structure. Following this, the specimens were mounted on frosted glass slides with 80% glycerol, with transparent nail enamel to coat the edges of the coverslip. The images were captured at x10 magnification. Image J software (NIH, Bethesda, MD, US) helped analyze the area in microns (μm2) of the initial and final size of each lesion.

Statistical Analysis

Data were analyzed using SPSS software for Windows (version 24.0; IBM Corp., Armonk, NY, USA). The paired t-test and one-way analysis of variance (ANOVA) were used to make comparisons in mean lesion depth before and after, between and across the groups, respectively. For all tests, a p-value of <0.05 was considered statistically significant.

## Results

**Figure 1:**
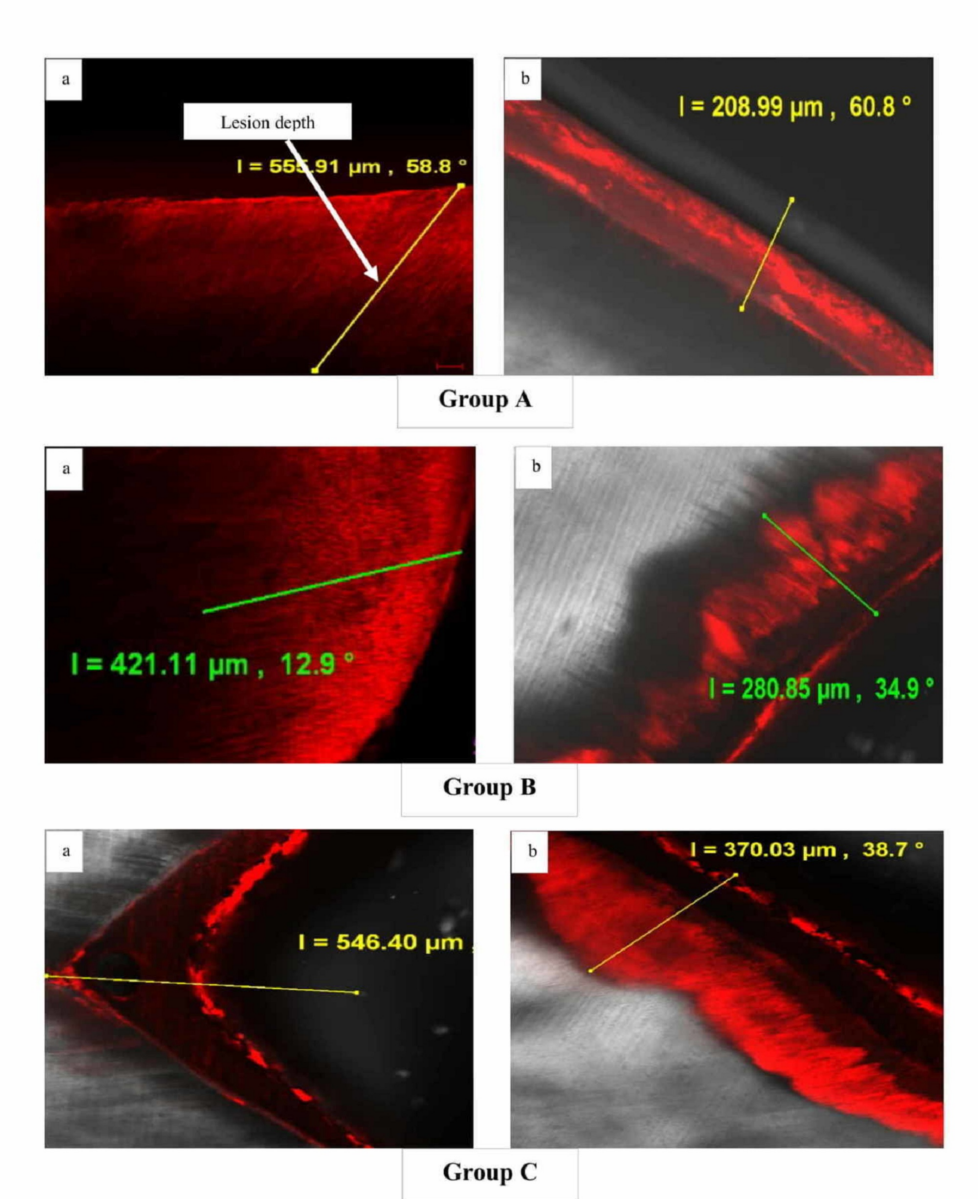
Representative confocal laser scanning microscopic images [5x] of groups A, B, and C (a) before and (b) after pH cycling

Figure 1 shows representative confocal images of enamel specimens for each group (A, B, and C) before and after pH cycling. At baseline, no significant difference was observed across the groups, though the mean lesion depth values were found to be higher for specimens in Group C (552.44 ± 42.09) followed by Group B (543 ± 33.76) and Group A (535.66 ± 25.22) (p = 0.591, one-way ANOVA). Following pH cycling for seven days, specimens from all groups showed significant remineralization, with a reduction in the mean lesion depth values (p<0.001, one-way ANOVA).

On comparison between groups, though a significant reduction in lesion depth was evident in all the groups, the maximum reduction (increased remineralization) was observed for specimens treated in Group A (294.98 ± 37.42), closely followed by Group B (280.78 ± 46.33) and least in Group C (177.13 ± 44.64) (Table 1).

**Table 1:** Difference in mean lesion depth between groups before and after pH cycling *Paired t-test

Groups	Lesion depth (Mean ± SD)		Mean difference	t-value	p-value
	Baseline*	Post-cycle*			
Group A (Tri-calcium phosphate dentifrice)	535.66 ± 25.22	240.68± 26.68	294.98 ± 37.42	23.64	0.000*
Group B (Amine fluoride dentifrice)	543 ± 33.76	262.21 ± 28.04	280.78 ± 46.33	18.18	0.000*
Group C (Theobromine dentifrice)	552.44± 42.09	375.30 ± 15.37	177.13 ± 44.64	11.90	0.000*

**Figure 2:**
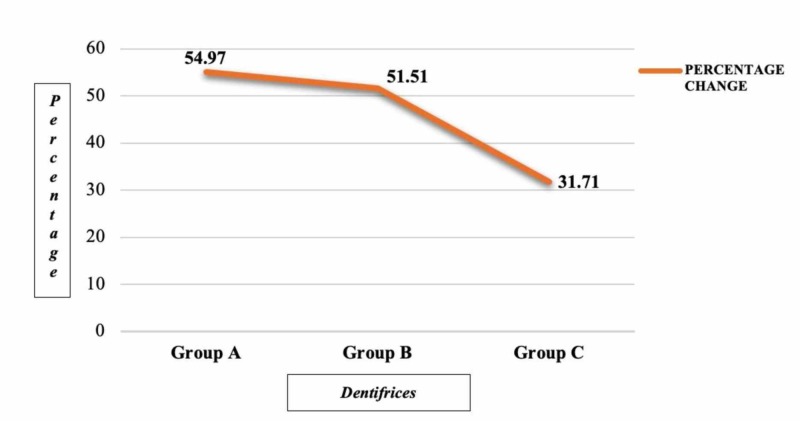
Percentage change in lesion depth among three groups after pH cycling

Figure 2 indicates the percentage change of lesion depth among three groups after pH cycling The percentage change in lesion depth was reported highest for specimens treated with NaF + f-TCP containing dentifrice, followed by amine fluoride and theobromine, respectively.

## Discussion

The dynamic nature of caries makes its management time-specific and critical [18]. With early diagnosis, the disease can be reversed in its initial stages, but it is irreversible and destructive after cavitation. An array of treatment strategies have been used for the early intervention of carious lesions, from surgical techniques to non-invasive methods involving biological agents.

The tooth surface is exposed to a constant environment of DML and RML resulting from microbial attack or dietary acids. DML results in loss of calcium and phosphorous from hydroxyapatite (HA) crystals. Reversal of the latter can be achieved if the pH is neutralized and there exist adequate calcium and phosphate ions in the immediate oral environment, thereby aiding in the rebuilding of dissolved HA crystals (RML) [3]. Hence, equilibrium must exist between the two processes to prevent the breakdown of the HA latticework, which results in cavities.

Newer strategies aiming to halt or reverse the progression of non-cavitated, demineralized lesions involve the topical use of RML agents. These agents provide an environment favorable for lesion repair and increase enamel resistance to acid challenge. In-vitro systems are those commonly used to understand the effects of such agents on carious processes. These systems are used owing to the low cost and time consumed as compared to other testing methods. In addition, these models help perform experiments in a controlled environment [19].

The present study compares the remineralization potential of three dentifrices using a pH cycling model. The latter model has been the choice over traditional pH cycling models and has demonstrated significant reliability. All specimens in the study were subjected to standard pH cycling consisting of seven days. This included 3 h of demineralization twice daily, with 2 h of remineralization in between. To replicate early, mid-day, and bedtime brushing, dentifrices were applied thrice daily. The advantage of using such a model is that, to a larger extent, it stimulates the real-life conditions leading to the development of caries [20].

Confocal laser scanning microscopy was utilized in the present study to monitor DML and RML patterns. Images obtained through this are similar to those from scanning electron microscope. Further, it helps reduce blur and provides high-quality images of increased resolution, the common issues often associated with images from standard fluorescence microscopy [21].

Over the years, fluorides containing dentifrices have been the usual choice for arresting caries. However, reports indicate that decline in caries may be at the verge or even in reversal, with increasing levels in some areas [22]. Thus, a constant need for the development of newer biomaterials, which act as an adjunct to the existing fluorides or can act individually as agents for arresting caries and promoting remineralization is advocated. The three different remineralization agents used for comparison in this study were those containing sodium fluoride with tri-calcium phosphate, amine fluoride, and theobromine. All RML agents used in the study reported increased efficacy in boosting enamel remineralization, even though they varied in their composition and mechanism of action.

Tricalcium phosphate (TCP) is a hybrid material created by milling beta-tricalcium phosphate (ß-TCP) and sodium lauryl sulfate [23]. In the present study, a greater degree of remineralization was evident in the group treated with f-TCP + NaF dentifrice and was similar to findings reported by Karlinsey et al. [24-25]. The results were also comparable to another study, where f-TCP + NaF provided a superior remineralization effect as compared with a 5000 ppm fluoride and casein phosphopeptide-amorphous calcium phosphate (CPP-ACP) dentifrice [26]. This increased remineralization potential may be the functionalized calcium phosphate system that is stable in the aqueous environment and does not affect the fluoride activity added in the dentifrices, which thereby boosts enamel surface strength [23].

Dentifrices containing amine fluorides demonstrate greater anti-cariogenic properties, considering the presence of fluoride, and the amine component responsible for the antiplaque effect, which allows for the accumulation and sustained fluoride release close to the tooth surface [27]. In this study, the group treated with amine fluoride reported a significant reduction in lesion depth thereby promoting remineralization but was less effective when compared to TCP but comparatively more effective than theobromine dentifrice.

Theobromine, previously known as “xantheose,” is a major constituent in the cocoa bean [11] and is the newer available remineralizing agent. The cariostatic property of theobromine accounts for the presence of two types of substances: one which exhibits anti-glucosyltransferase and the other anti-bacterial activity [12]. In the present study, theobromine was found to be effective in remineralizing carious lesions but less so when compared to TCP and amide fluoride dentifrice. The results were comparable to other studies, where theobromine remineralized carious lesions and increased enamel microhardness, providing increased resistance to acid attack [13,28]. On the contrary, in one study, theobromine failed to provide any anti-caries effect [29]. Thus, the available evidence is suggestive that theobromine containing dentifrices demonstrates significant remineralizing potential. However, the clinical recommendation for the use of theobromine requires further studies to be carried out in-situ and clinical trials involving a group of high-risk participants. Another positive factor for the use of theobromine as an alternative to fluoride is the relative ease at which it is absorbed and metabolized in the human body [15], making it free from the adverse side-effects (dental fluorosis, tooth discoloration, and gastric irritation with high doses) related with use of fluorides [5].

Limitations are inevitable with in-vitro studies such as the present study. These include the lack of a real-life environment, which would be present in the oral cavity. Moreover, the specimens in the pH cycle are subjected to rigorous remineralization and demineralization, which are more aggressive than the usual acid attacks that a tooth is exposed to in the oral cavity. Another possible limitation is due to the variation in sample selection and allocation, as some teeth might pose greater susceptibility than others to demineralization due to the age of the donor and exposure to environmental factors, thus influencing the outcome. Hence, it is apparent that no in-vitro model can be a realistic substitute for the conditions that prevail in the oral cavity.

## Conclusions

A variety of new-age remineralizing agents are available in the market. Thus, a need to understand the benefits and efficacy of these products for its recommendation for use in clinical practice is vital. Within the limitations of this experimental study, all dentifrices were effective in remineralizing artificial carious lesions. Although promising results were evident with the remineralizing ability of theobromine, on comparison to dentifrices containing f-TCP+NaF and amine fluoride, it was found to be the least effective.
